# Supplementary material for: Personalizing image enhancement for critical visual tasks: improved legibility of papyri using color processing and visual illusions
Source: Int J Doc Anal Recognit. 2021 Dec 27;25(2):129–60. doi: 10.1007/s10032-021-00386-0 (PMC9106648; doi:10.1007/s10032-021-00386-0)
Supplement: Supplementary file 1 — Supplementary material 1 (pdf 16858 KB) [file 10032_2021_386_MOESM1_ESM.pdf]

Supplementary Material

Presented herein are the ninety images used in the evaluation of papyri legibility enhancement experiment. The methods are described in the article, Sections 4 Methods and 5.1 Algorithms.

TSVP →

Vlad Atanasiu, Isabelle Marthot-Santaniello (2021) — "Personalizing image enhancement for critical visual tasks: improved legibility of papyri using color processing and visual illusions", Journal on Document Analysis and Recognition (IJDAR).

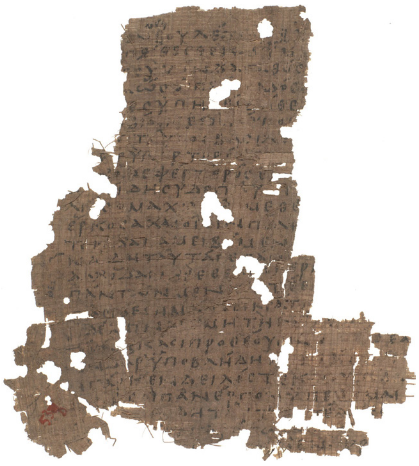

original

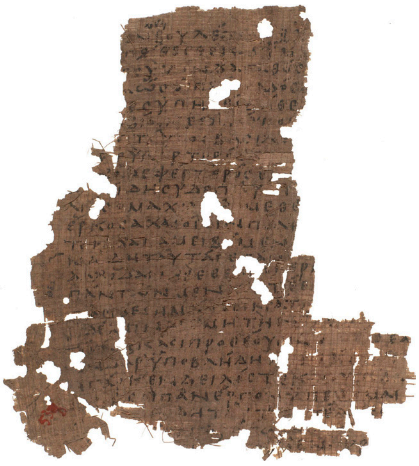

stretchlim

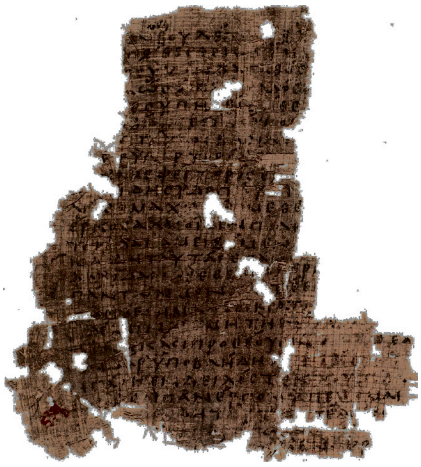

histeq

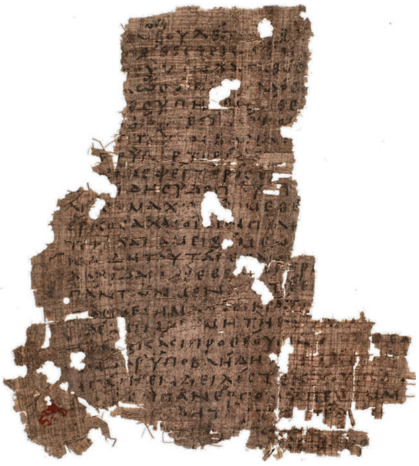

adapthisteq

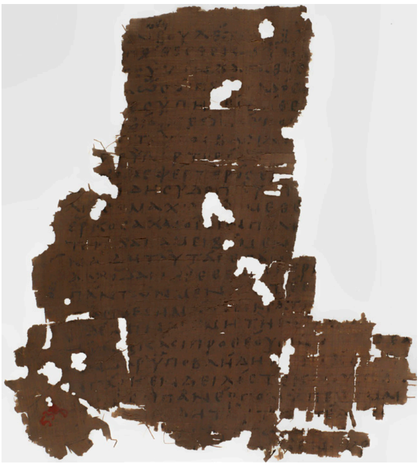

locallapfilt

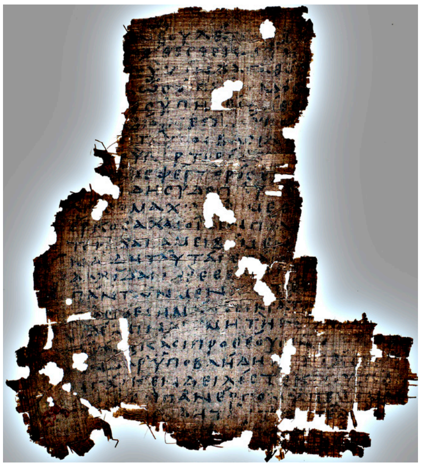

retinex

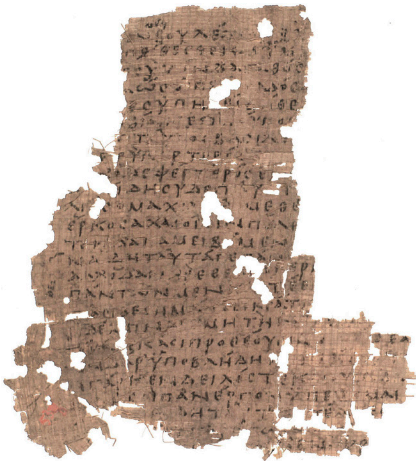

lsv

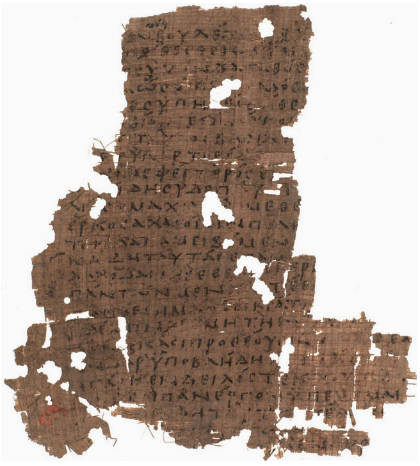

vividness

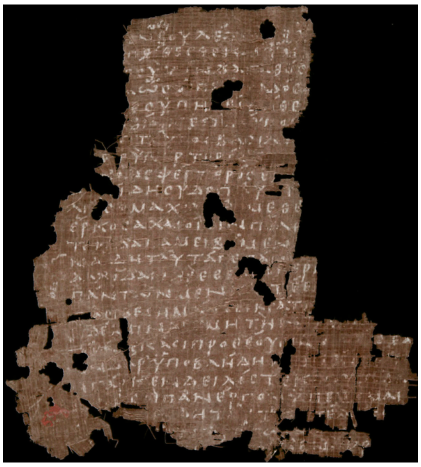

neglsv

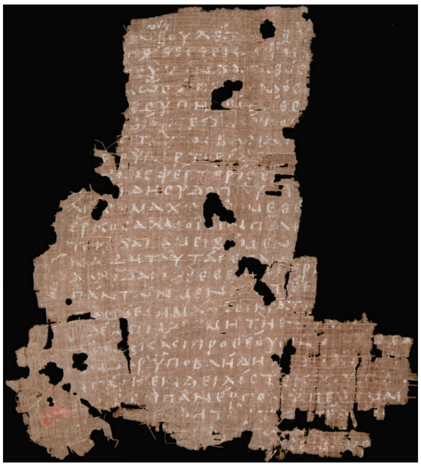

negvividness

columbia.apis.p367.f.0.600

Credit of original papyrus reproduction: Columbia University Library, CC BY-NC 3.0

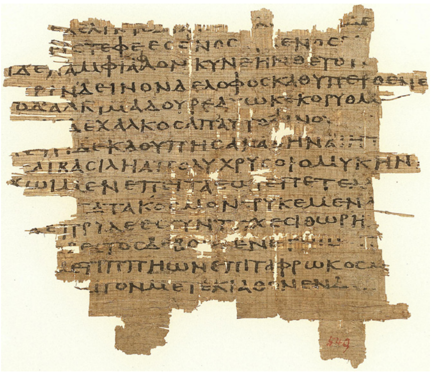
original

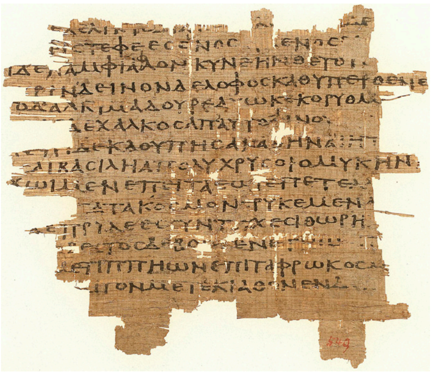
stretchlim

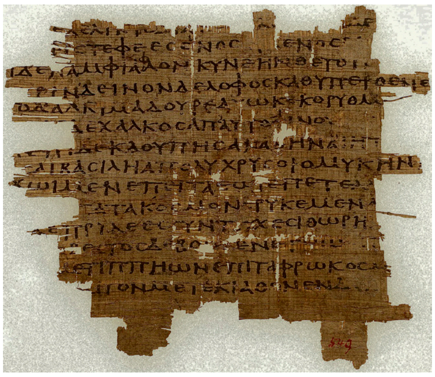
histeq

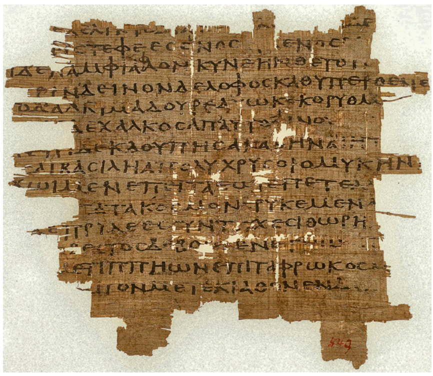
adapthisteq

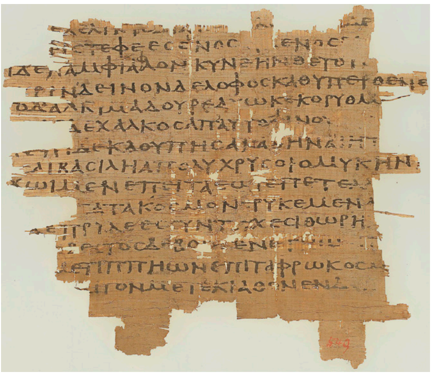
locallapfilt

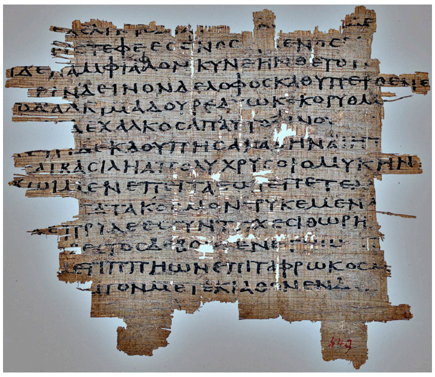
retinex

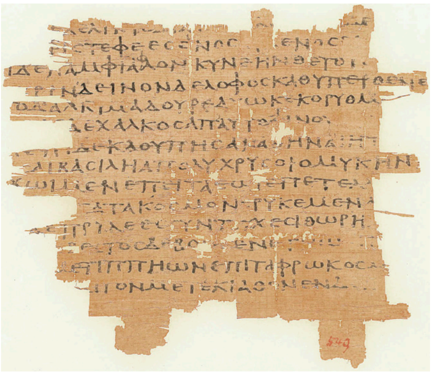
lsv

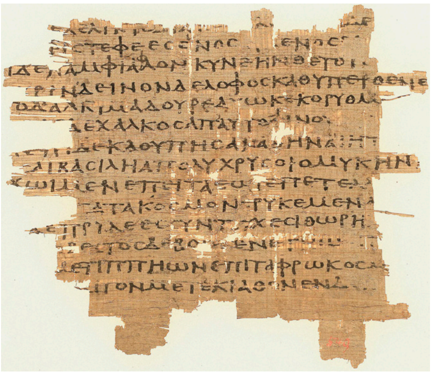
vividness

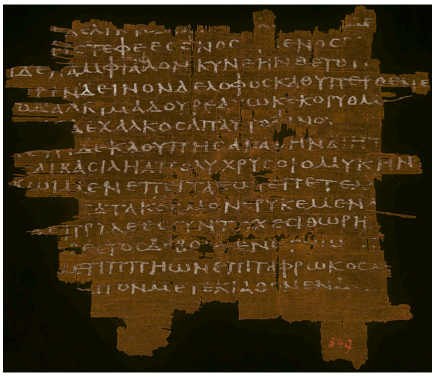
neglsv

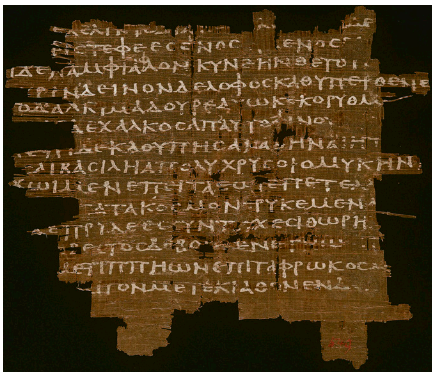
negvividness

P.Corn. Inv. MSS.A 101. XIII

Credit of original papyrus reproduction: University of Michigan Library, Public Domain Mark 1.0

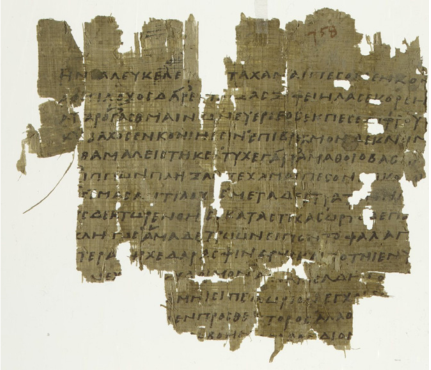

original

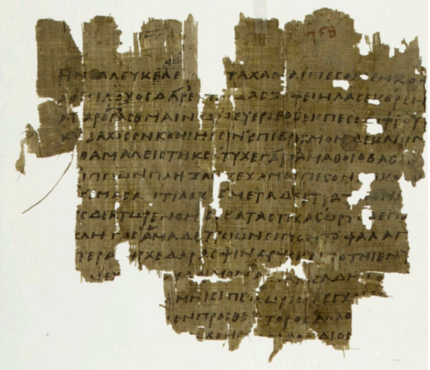

stretchlim

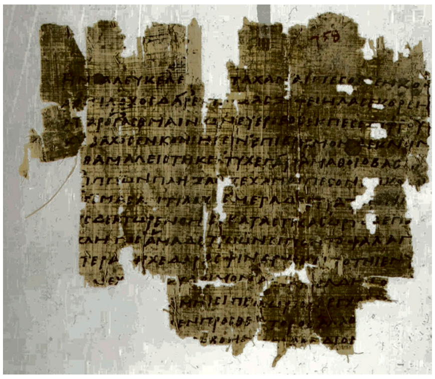

histeq

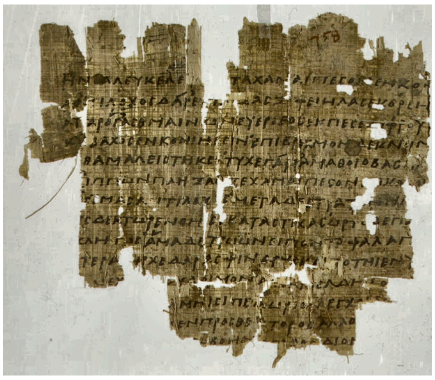

adapthisteq

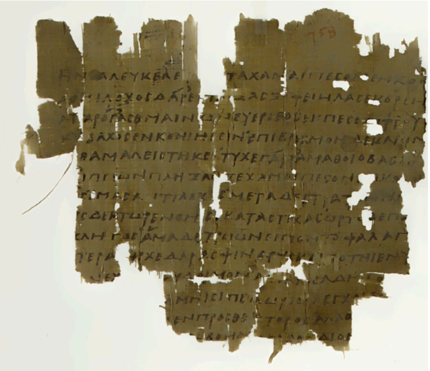

locallapfilt

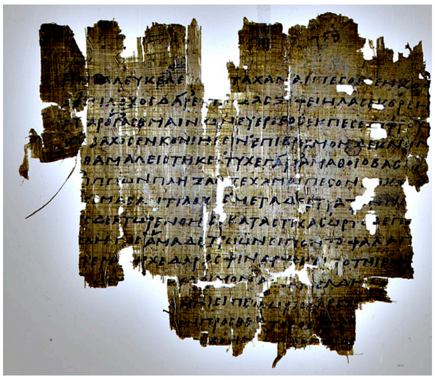

retinex

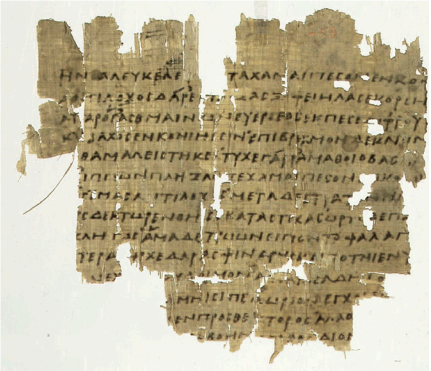

lsv

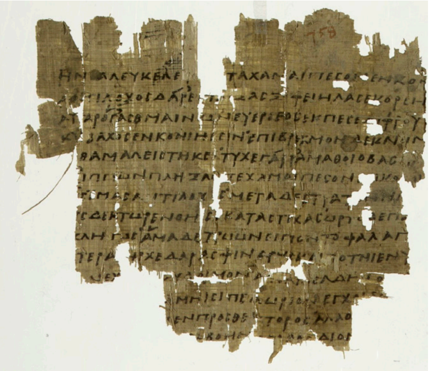

vividness

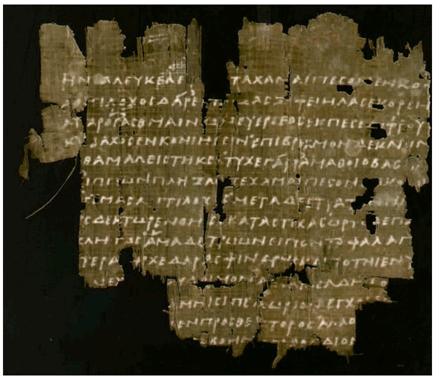

neglsv

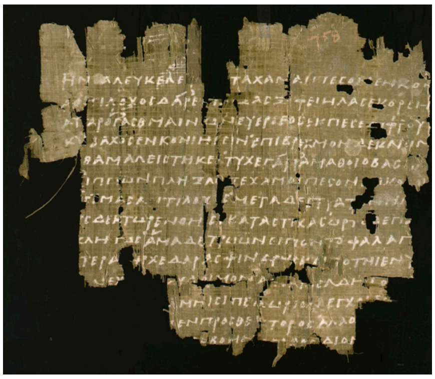

negvividness

P.CtYBR inv. 69

Credit of original papyrus reproduction: Yale University Library, Public Domain Mark 1.0

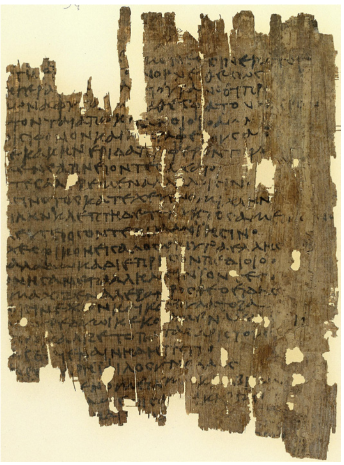

original

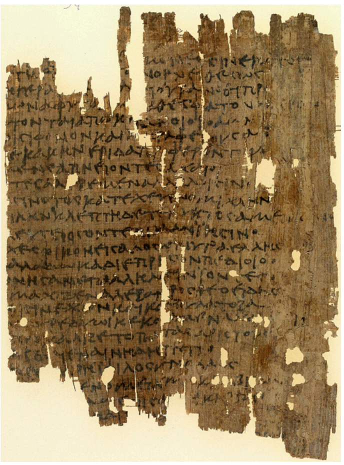

stretchlim

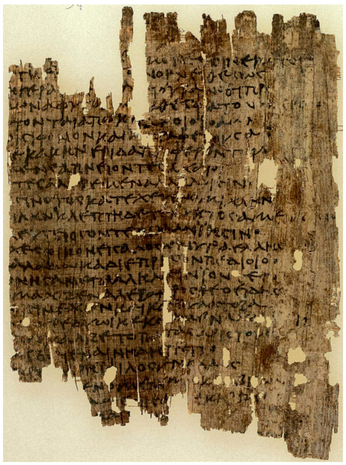

histeq

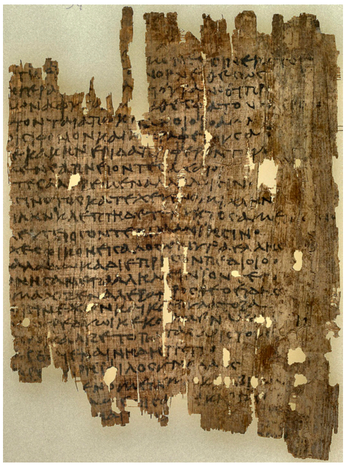

adapthisteq

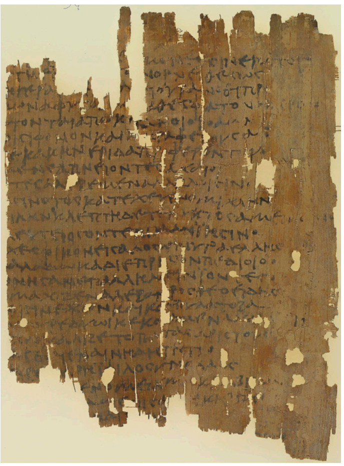

locallapfilt

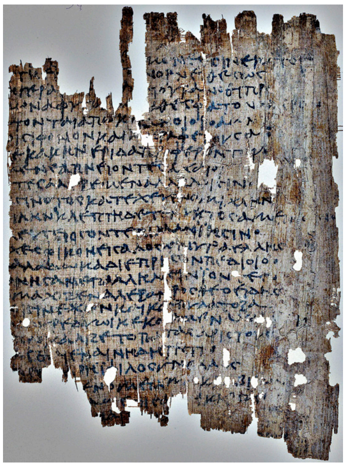

retinex

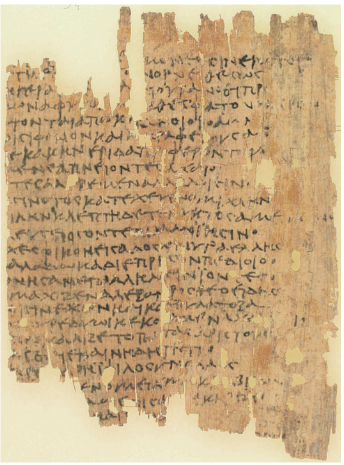

lsv

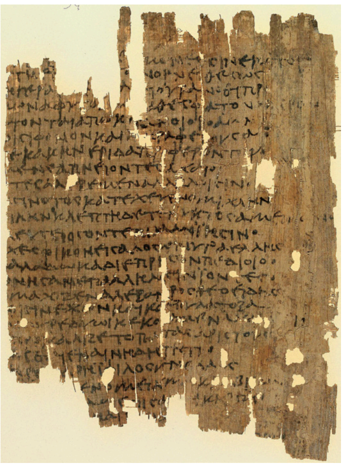

vividness

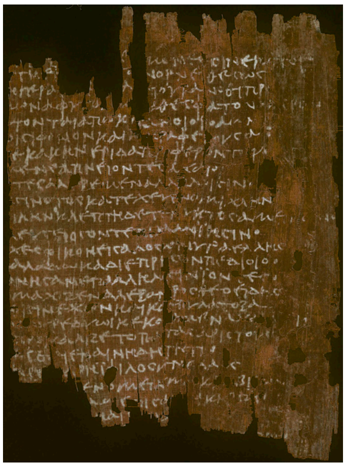

neglsv

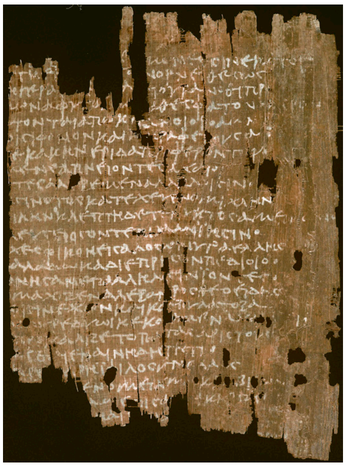

negvividness

P.Mich.inv.1318v

Credit of original papyrus reproduction: University of Michigan Library, Public Domain Mark 1.0

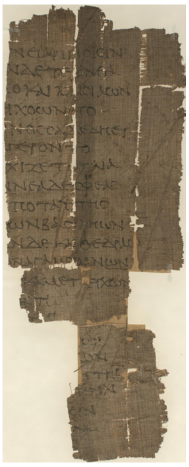

original

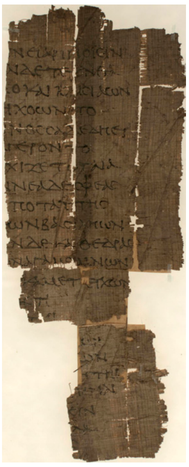

stretchlim

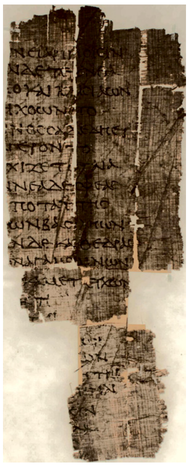

histeq

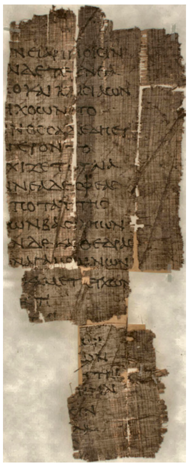

adapthisteq

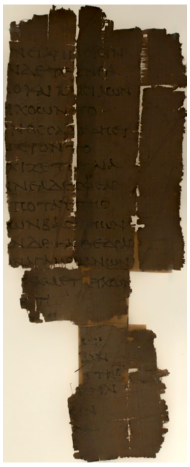

locallapfilt

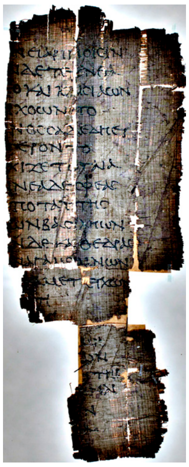

retinex

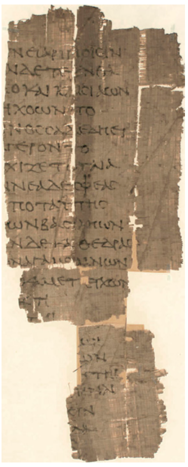

lsv

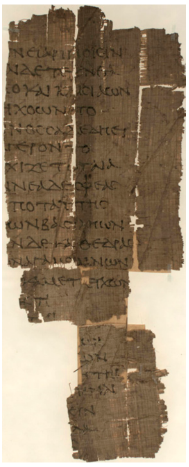

vividness

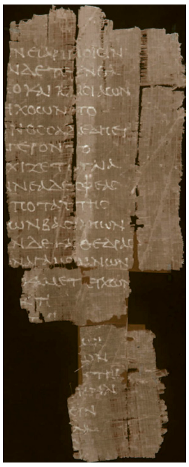

neglsv

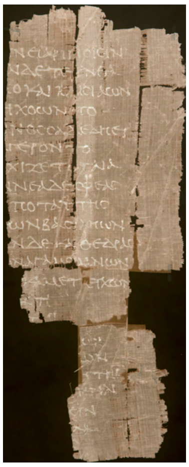

negvividness

P.Mich.inv.2755

Credit of original papyrus reproduction: University of Michigan Library, Public Domain Mark 1.0

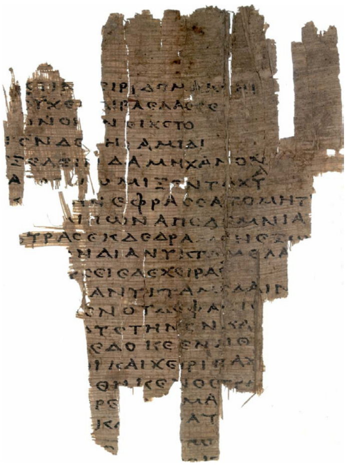

original

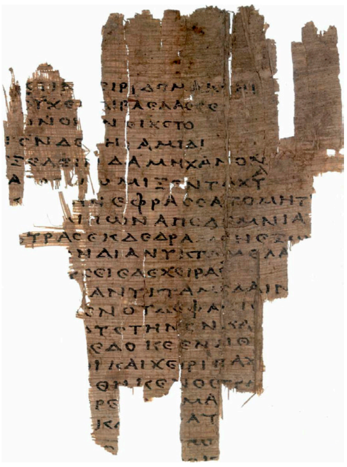

stretchlim

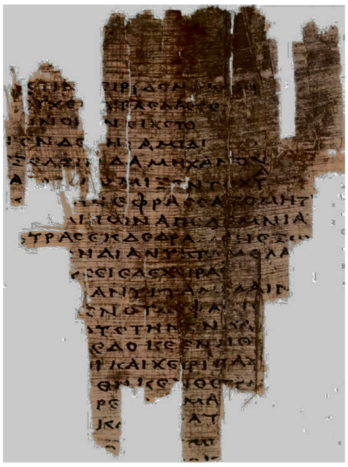

histeq

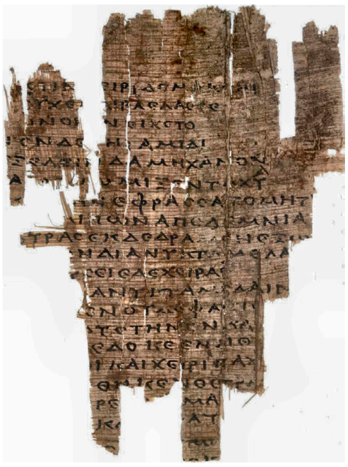

adapthisteq

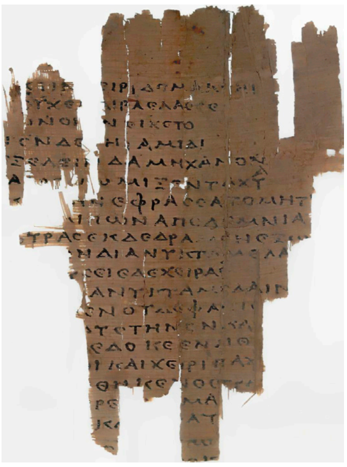

locallapfilt

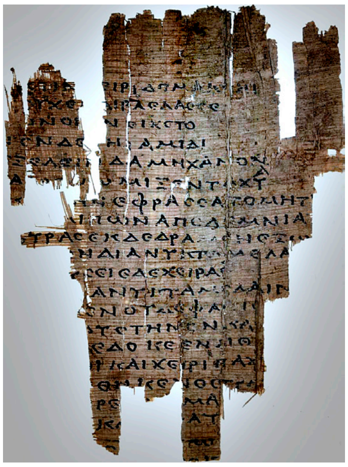

retinex

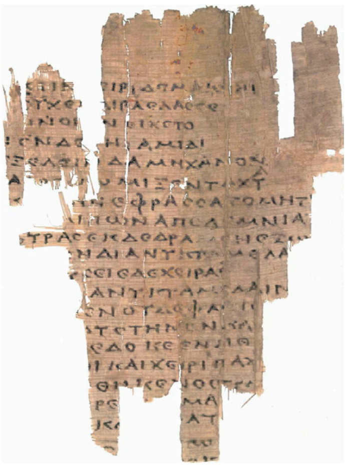

lsv

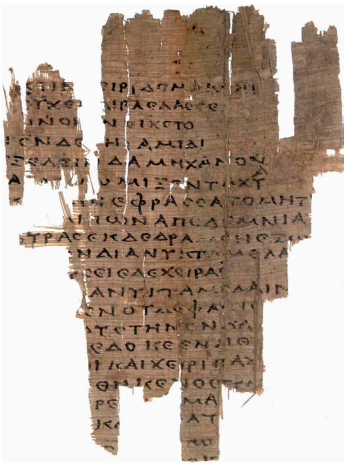

vividness

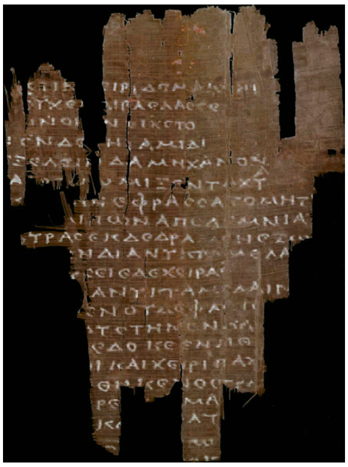

neglsv

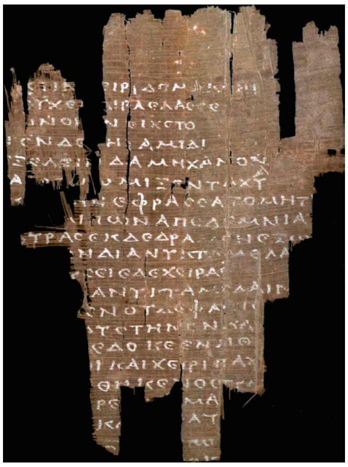

negvividness

P.Oxy.XXII 2309

Credit of original papyrus reproduction: Courtesy of The Egypt Exploration Society and the University of Oxford Imaging Papyri Project

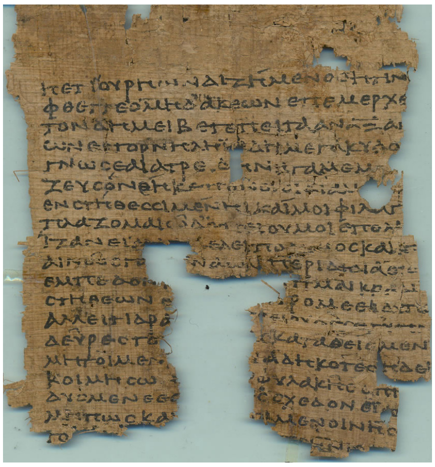
original

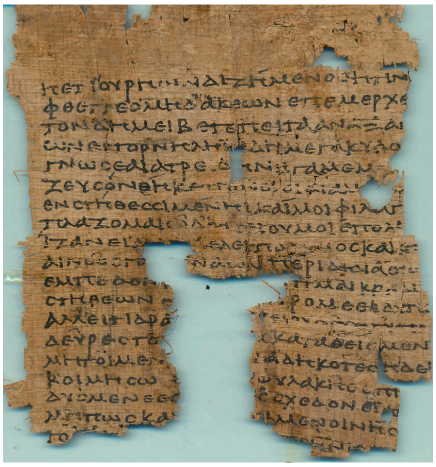
stretchlim

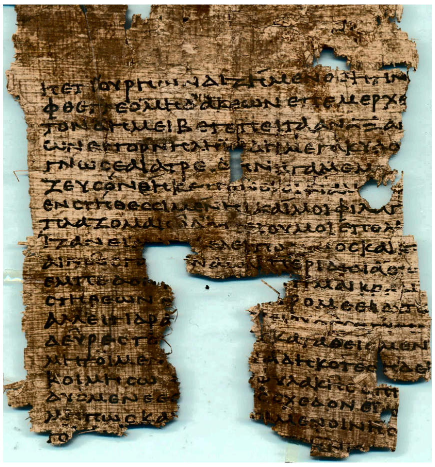
histeq

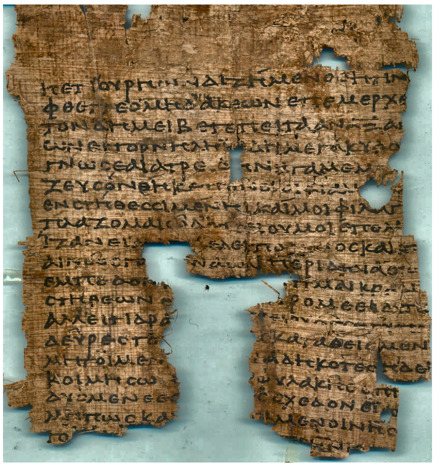
adapthisteq

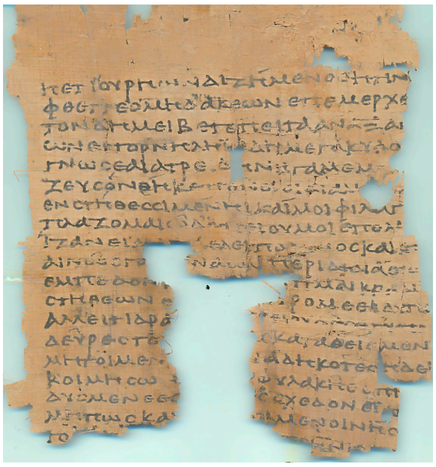
locallapfilt

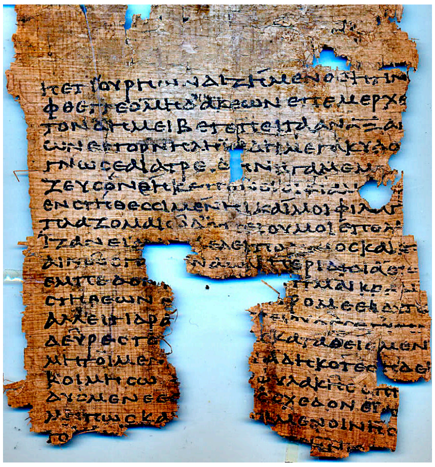
retinex

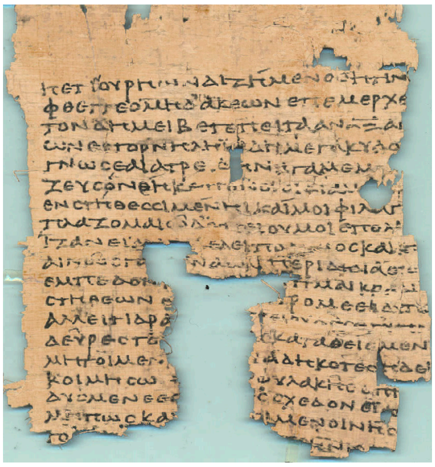
lsv

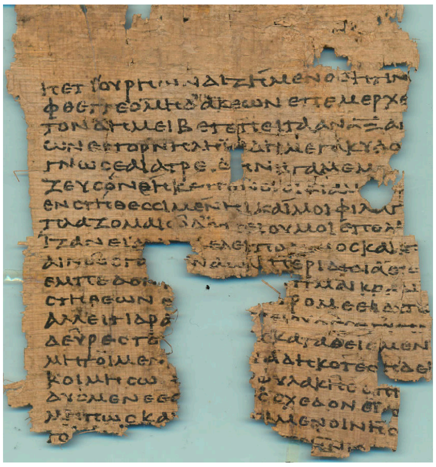
vividness

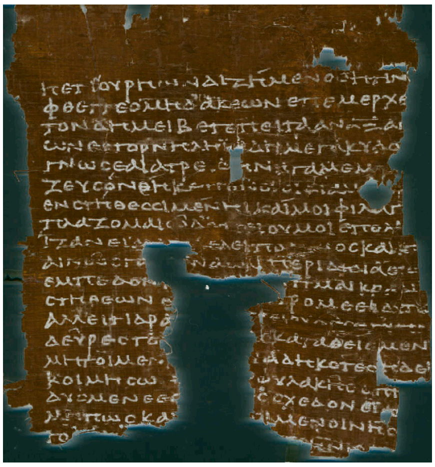
neglsv

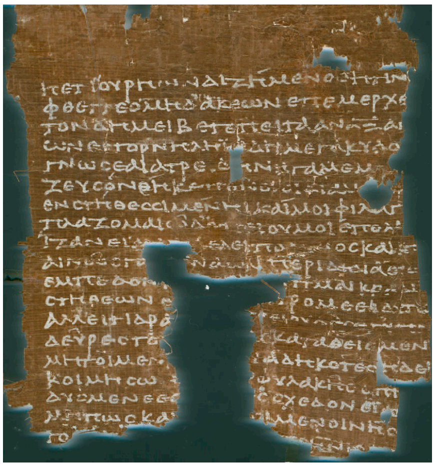
negvividness

PSI XII 1274 r

Credit of original papyrus reproduction: Istituto Papirologico Vitelli, by permission

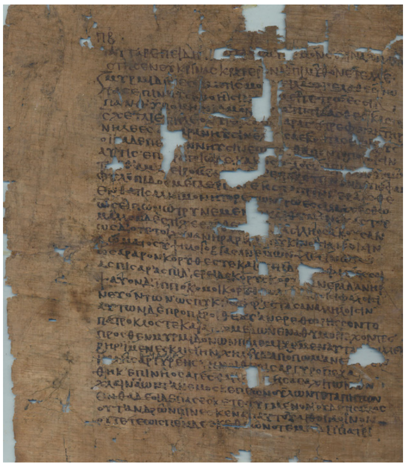

original

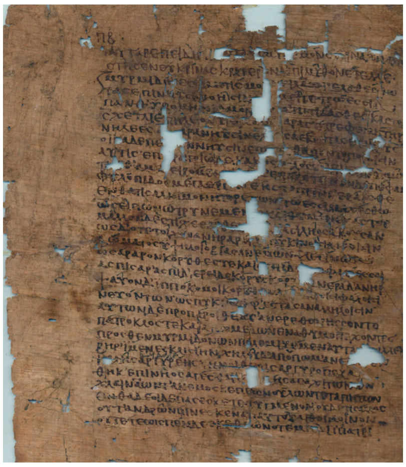

stretchlim

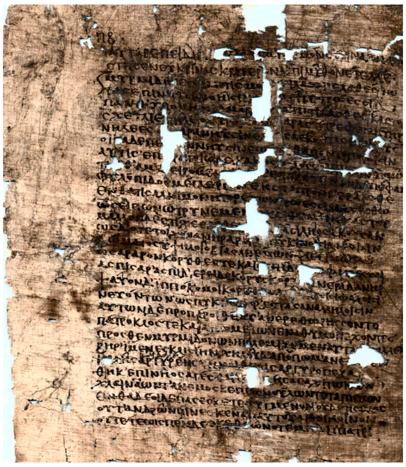

histeq

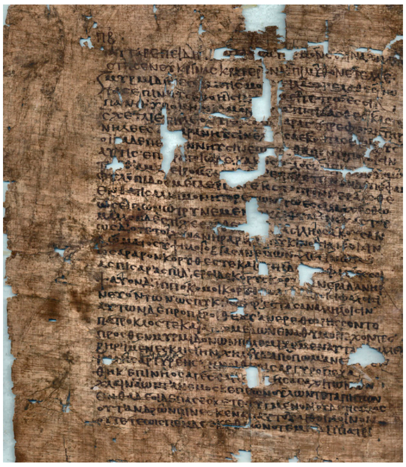

adapthisteq

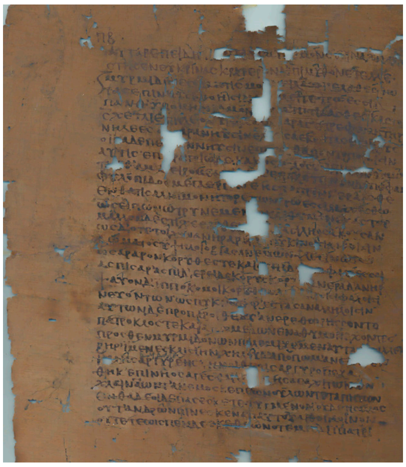

locallapfilt

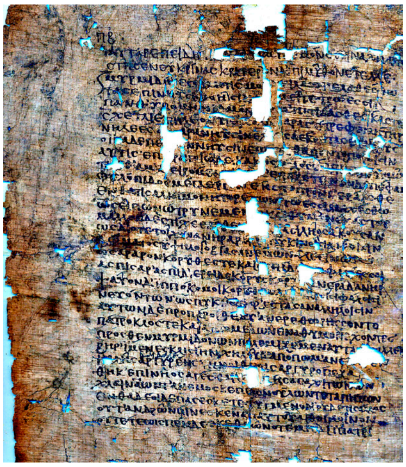

retinex

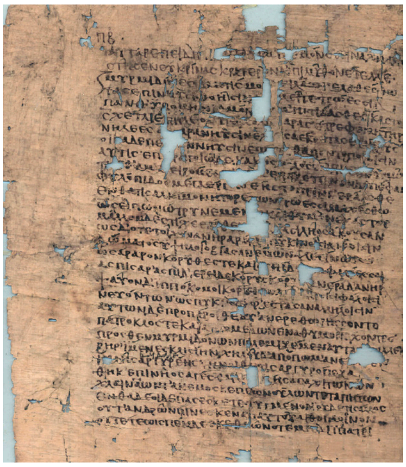

lsv

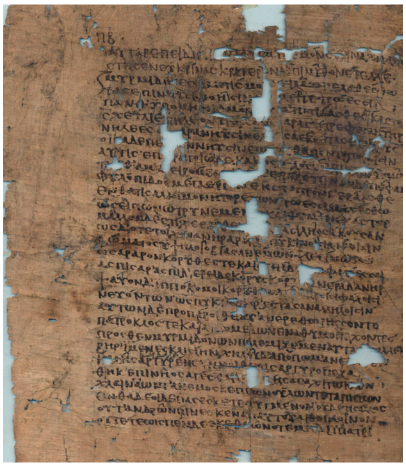

vividness

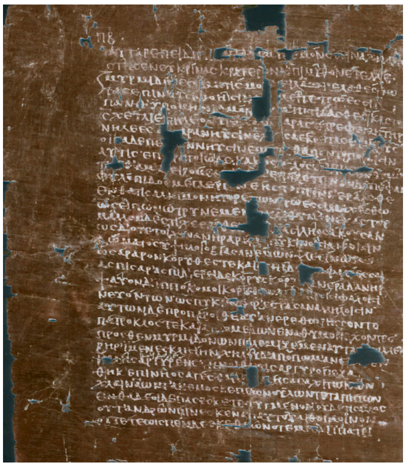

neglsv

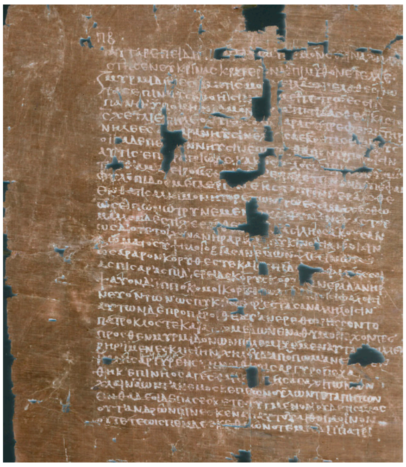

negvividness

PSI XIII 1298 (15a) r 1

Credit of original papyrus reproduction: Istituto Papirologico Vitelli, by permission

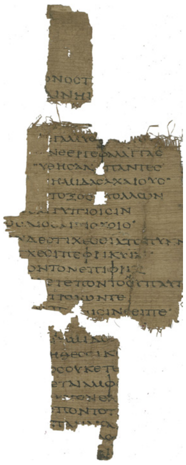

original

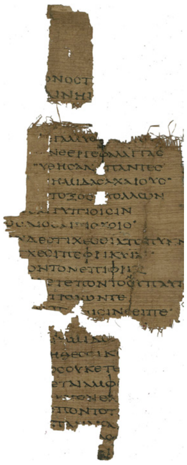

stretchlim

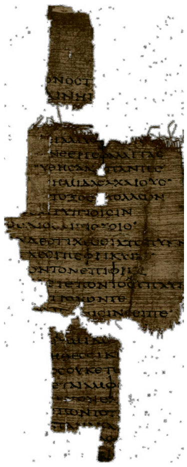

histeq

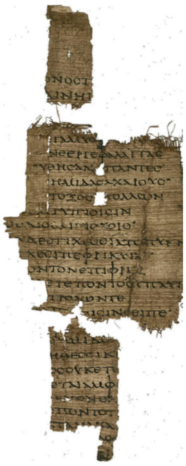

adapthisteq

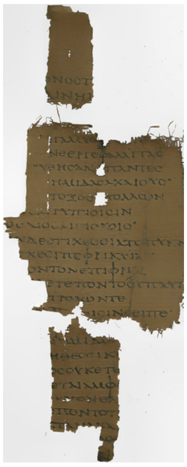

locallapfilt

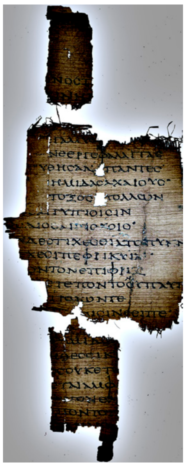

retinex

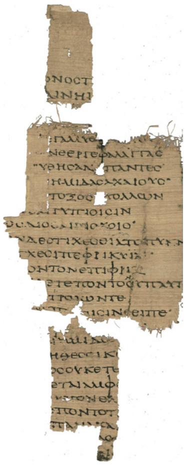

lsv

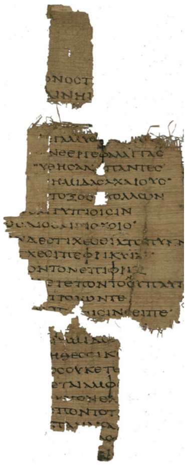

vividness

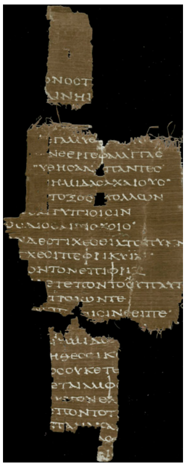

neglsv

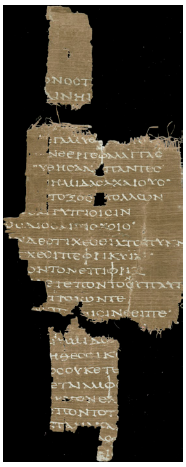

negvividness

PSI XIV 1376 r

Credit of original papyrus reproduction: Istituto Papirologico Vitelli, by permission
